# Rhodium-Complex-Functionalized and Polydopamine-Coated CdSe@CdS Nanorods for Photocatalytic NAD^+^ Reduction

**DOI:** 10.1021/acsanm.1c02994

**Published:** 2021-12-02

**Authors:** Marcel Boecker, Mathias Micheel, Alexander K. Mengele, Christof Neumann, Tilmann Herberger, Tommaso Marchesi D’Alvise, Bei Liu, Andreas Undisz, Sven Rau, Andrey Turchanin, Christopher V. Synatschke, Maria Wächtler, Tanja Weil

**Affiliations:** † Department for Synthesis of Macromolecules, Max Planck Institute for Polymer Research, 55128 Mainz, Germany; ‡ Department of Functional Interfaces, Leibniz Institute of Photonic Technology, 07745 Jena, Germany; § Institute of Inorganic Chemistry I, Ulm University, 89081 Ulm, Germany; ∥ Institute of Physical Chemistry, Friedrich Schiller University Jena, 07743 Jena, Germany; ⊥ Abbe Center of Photonics (ACP), Albert-Einstein-Straße 6, 07745 Jena, Germany; # Institute of Materials Science and Engineering, Chemnitz University of Technology, 09125 Chemnitz, Germany; ∇ Otto Schott Institute of Materials Research, Friedrich Schiller University Jena, 07743 Jena, Germany

**Keywords:** photocatalysis, polydopamine, CdS nanorods, NAD^+^ reduction, photocatalytic system

## Abstract

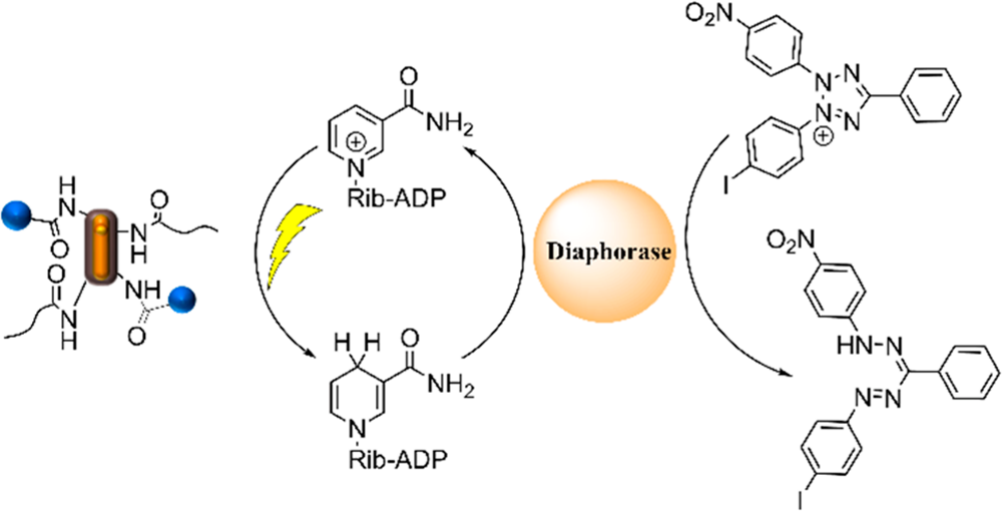

We report on a photocatalytic system consisting of CdSe@CdS nanorods coated with a polydopamine (PDA) shell functionalized with molecular rhodium catalysts. The PDA shell was implemented to enhance the photostability of the photosensitizer, to act as a charge-transfer mediator between the nanorods and the catalyst, and to offer multiple options for stable covalent functionalization. This allows for spatial proximity and efficient shuttling of charges between the sensitizer and the reaction center. The activity of the photocatalytic system was demonstrated by light-driven reduction of nicotinamide adenine dinucleotide (NAD^+^) to its reduced form NADH. This work shows that PDA-coated nanostructures present an attractive platform for covalent attachment of reduction and oxidation reaction centers for photocatalytic applications.

In recent decades, the field of photocatalysis has greatly expanded to meet increasing energy demands.^1^ Inspired by nature’s photosynthesis, photocatalysis uses photons as the energy source to fuel chemical reactions, thereby making use of the ultimate renewable energy source, the sun.^2^ These photocatalytic reactions transform chemicals into more useful, high-value compounds or create molecules with higher energy density for energy storage.^1,2^

Semiconductor nanostructures based on cadmium chalcogenides have been explored as efficient photosensitizers for performing a range of photocatalytic reactions.^3^ In particular, CdSe@CdS dot-in-rod nanostructures have shown high activity for light-driven hydrogen generation in aqueous solution when connected to reaction centers such as metal nanoparticles,^4^ redox-active enzymes,^5^ or transition metal complexes.^6^ In these structures, charge separation is very efficient because the photogenerated hole localizes in the CdSe core and the electron is transferred to the catalytic center, supporting charge accumulation at the reaction center to drive multielectron reactions.^4^ Unfortunately, these photosensitizers easily undergo photo-oxidation if the holes remaining in the semiconductor nanostructure are not efficiently quenched, which limits their long-term usage.^7^ Therefore, the addition of sacrificial electron donors is necessary to stabilize catalytic systems. The choice of quencher determines the rate and efficiency of hole quenching and, as a consequence, the catalytic efficiency and long-term stability increase.^8^

The highly cross-linked melanin-like biopolymer polydopamine (PDA)^9^ has been applied in several photocatalytic systems to improve their efficiency. It forms universal multifunctional coatings through a simple dopamine autoxidation process, which forms a highly adhesive polycatechol-based polymer.^10^ Moreover, PDA films reveal broad-band absorption and electron-donating properties^11^ and hence can act as a protective layer that reduces photo-oxidation.^11^ They also support electron transport toward, e.g., reaction centers, similar to natural photosystem II, either mediated via electron-accepting groups^12^ or by direct tunneling in the case of very thin layers.^11^ Previously, CdS/PDA/TiO_2_ core/shell nanoparticles have been achieved in which the PDA layer improved the photocurrent and photocatalytic performance because of enhanced light absorption and charge carrier mobility.^13^ Furthermore, PDA coatings increased the photostability of CdS semiconductors, which facilitated the transfer of electrons to quench holes and prevented oxidation by the formation of a strong coordination bond.^13,14^ Reported PDA coatings for photocatalytic systems make use of the interaction of PDA and semiconductor or metal nanoparticles.^15^ Additionally, the presence of multiple functional groups in PDA also allows for straightforward surface functionalization, e.g., by reaction of the quinones of PDA with amines in Michael addition or Schiff base reactions.^16,17^ This in turn offers the opportunity to functionalize PDA also with molecular catalysts via various functional groups. This stands in contrast to the functionalization of bare CdSe@CdS nanorods, which is possible only with limited anchoring groups, e.g., thiols or dithiocarbamates.^6^

Motivated by these promising photocatalysis results with nanorods and PDA, we designed a photocatalytic system consisting of CdSe@CdS nanorods as a photosensitizer coated by a highly cross-linked, protective, and functionalizable PDA biopolymer layer and equipped the system with a rhodium-based molecular catalyst, [(ipphCOOH)Rh(Cp*)Cl]Cl, where Cp* is pentamethylcyclopentadienyl and ipphCOOH is a functionalized 1*H*-imidazo[4,5-*f*][1,10]phenanthroline.

The newly established photocatalytic system was characterized for the photocatalytic reduction of nicotinamide adenine dinucleotide (oxidized form, NAD^+^) to NADH.^18^ NADH is a key redox compound in all living cells that is responsible for energy transduction, genomic integrity, life-span extension, and neuromodulation,^19^ and it plays a key role in enzymatic reductions. Given the high cost, stoichiometric usage, and chemical instability of NADH, there is substantial interest in NADH regeneration.^20^ Recently, several systems comprising a Rh-based catalyst have been proposed for the purpose of light-driven reduction of NAD^+^ in combination with either molecular chromophores in a homogeneous catalytic approach^18,21^ or quantum dots as light absorbers.^22,23^

By combining the photosensitizer and the catalytic reaction center in close contact in a nanoparticulate assembly by linking them to the PDA matrix, the system presented herein serves as a suspended heterogeneous catalytic system. In contrast to the many reported homogeneous systems for photocatalytic reduction of NAD^+^, our approach offers the advantage to recover the photoactive nanomaterial from the reaction solution by centrifugation. In addition, different types of catalyst could be attached by simply functionalizing the PDA shell.

The CdSe@CdS nanorods (NRs) (length = 43.8 ± 5.8 nm, width = 4.8 ± 0.4 nm; Figure S7) were synthesized by the seeded growth approach^24^ (with a CdSe seed diameter of 2.0 nm), which yields nanorods with a quasi-type-II band alignment.^25^ The NRs were then transferred into an aqueous medium via ligand exchange with mercaptoundecanoic acid (for details, see the Supporting Information (SI)).^26^ For the synthesis of the molecular catalyst, 1,10-phenanthroline-5,6-diones were first prepared according to the literature^27^ and then reacted with 4-formylbenzoic acid and ammonium acetate to afford the carboxylic acid-functionalized phenanthroline ligand 4-(1*H*-imidazo[4,5-*f*][1,10]phenanthrolin-2-yl)benzoic acid (ipphCOOH) (for details, see the SI). The molecular catalyst was generated by ligand exchange upon mixing of ipphCOOH with [Rh(Cp*)Cl_2_]_2_ (for details, see the SI). Subsequently, the multicomponent photocatalytic system was generated in a two-step procedure, as shown in Scheme 1. First, the prepared NRs were coated with PDA by autoxidation of dopamine in alkaline Tris buffer (0.1 M, pH 8.5) for 24 h. Then the PDA-coated nanorods (cNRs) were purified by centrifuge filtration (100 kDa cutoff) and redispersed in Milli-Q water, which was repeated three times.

**Scheme 1:**
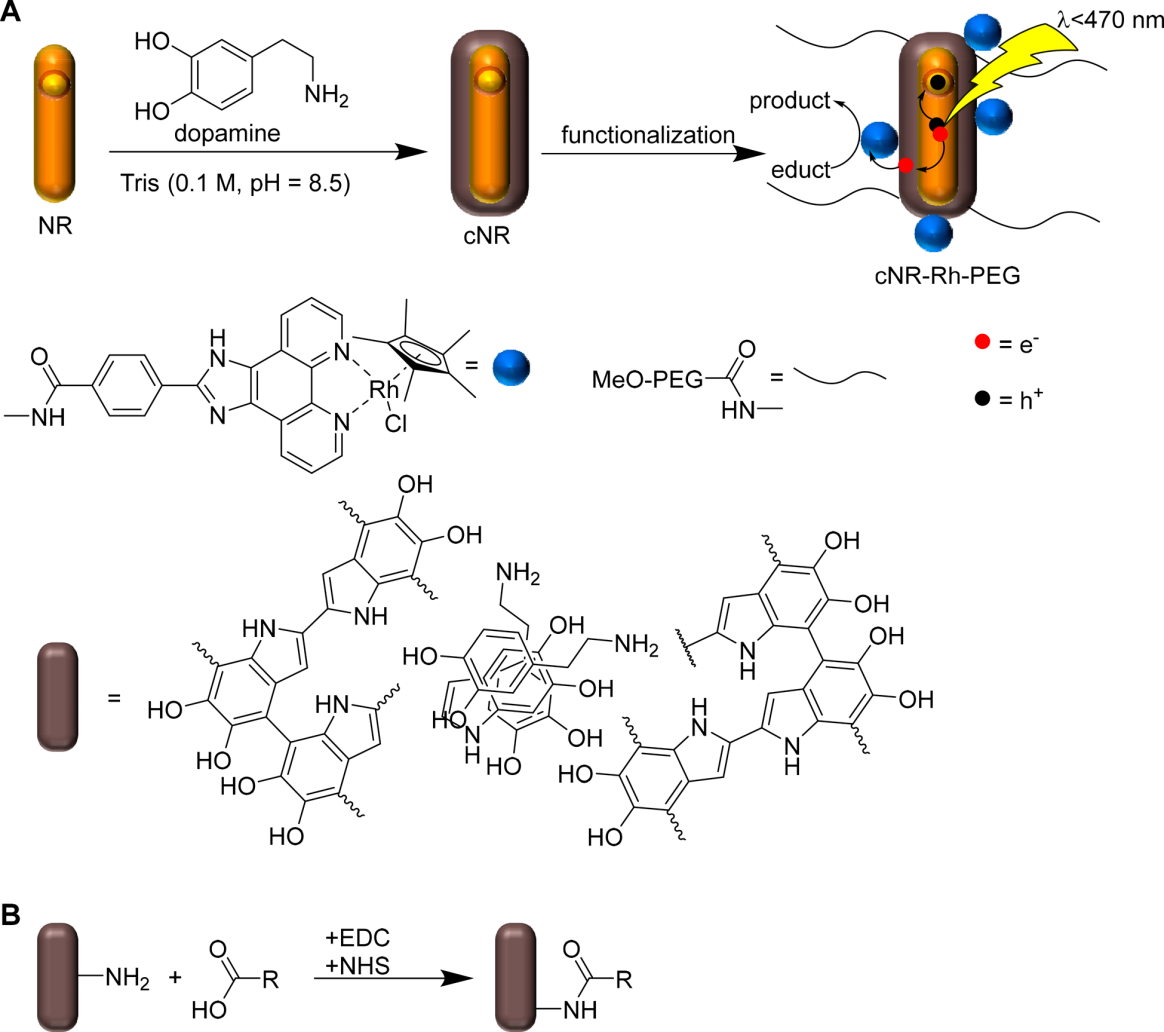
(A) Generation of the Photocatalytic System Based on CdSe@CdS Nanorods (NRs) Coated with PDA to Yield cNRs, Followed by Functionalization with Rh Catalysts and PEG (5 kDa) to Yield cNR-Rh-PEG; (B) Reaction Scheme for Amide Formation between the Free Amine Functionalities of PDA and Carboxylic Acid Functionalities of the Rh Catalyst and PEG

The as-synthesized NRs show a lattice spacing of 0.33 nm for the (002) plane (Figure 1B), which is characteristic of CdSe@CdS NRs because of the preferred growth along this facet.^24^ Furthermore, the X-ray diffraction pattern of the NRs confirms a wurtzite crystal structure (Figure S8). The PDA coating yielded a very thin shell (<5 nm) covering the NRs, as imaged by high-resolution transmission electron microscopy (HR-TEM) (Figure 1B). Such thin PDA shells have previously been formed on peptide nanofibers, which imparted additional functionalities for surface modification.^28^ PDA provides characteristic reduction properties,^10^ and the new PDA layer on the cNRs reduced HAuCl_4_ to elemental gold nanoparticles by a published procedure.^29^ The formation of gold nanoparticles was observed only on cNRs but not on uncoated NRs (Figure S9), which additionally proves the successful coating with PDA. However, these gold nanoparticles are not present in the final cNR-Rh-PEG photocatalytic system.

**Figure 1:**
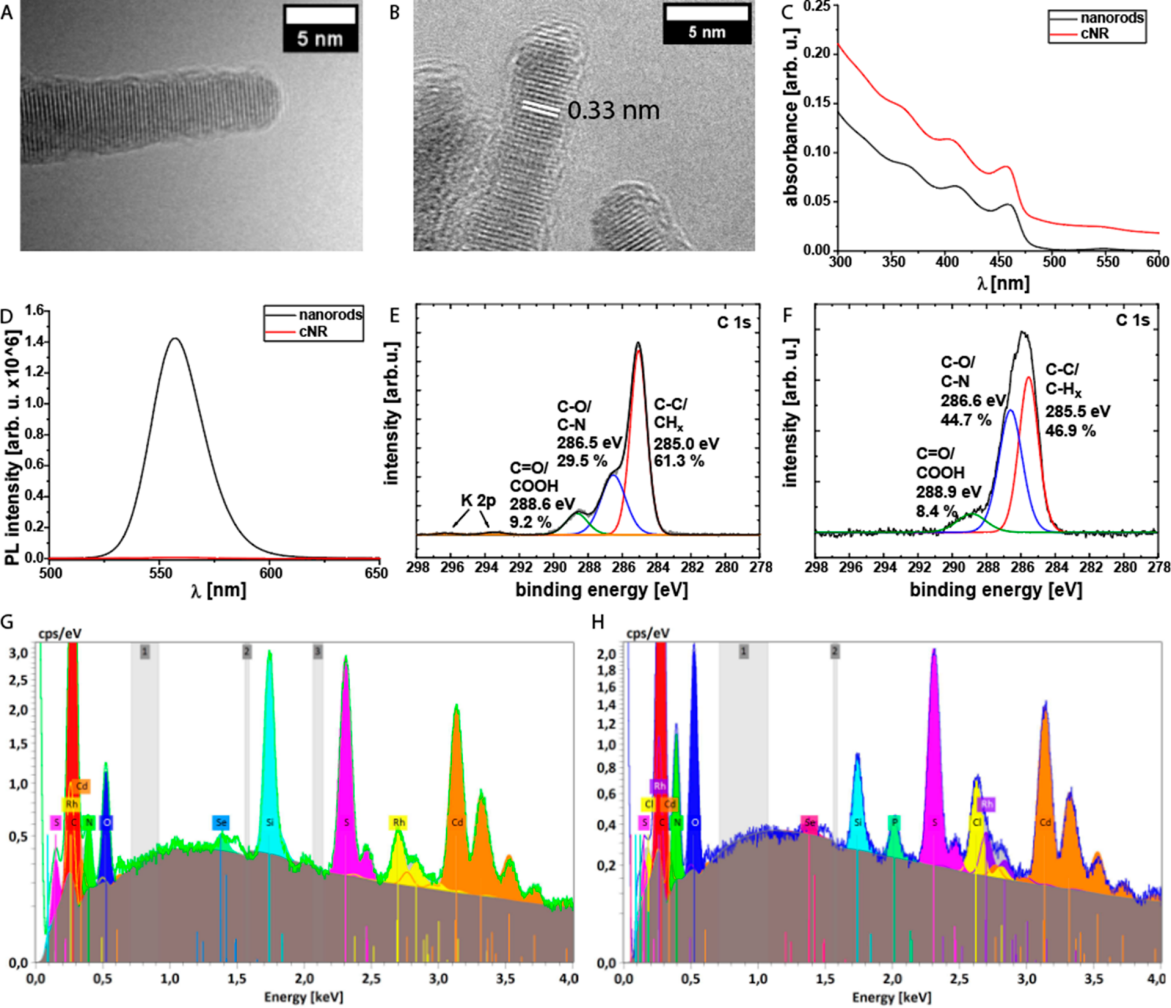
(A, B) TEM images of (A) pure NRs and (B) cNRs. (C) Absorption spectra of bare NRs and cNRs functionalized with PEG. (D) Photoluminescence spectra (λ_ex_ = 450 nm) of bare NRs and cNRs (grafted with PEG for better colloidal stability) in water. (E, F) High-resolution C 1s XP spectra of (E) cNRs and (F) cNR-Rh-PEG. (G, H) EDX spectra of cNR-Rh-PEG (G) before and (H) after irradiation.

Surface coating strongly affected the optical properties of the cNRs. The absorption spectrum of uncoated NRs is characterized by CdS rod absorption below 460 nm and CdSe absorption at 550 nm (Figure 1C), while cNRs revealed an extended absorption over the entire visible spectrum due to the broad absorption of the PDA shell.

Moreover, the photoluminescence (PL) quantum yield (QY) of cNRs indicates a very significant quenching effect of the QY induced by the PDA layer. The PL of NRs (QY = 0.14) was reduced about 200-fold after coating, with an estimated QY for cNRs on the order of 0.001 (Figure 1D). In general, the PL QY is a measure of radiative exciton recombination efficiency in NRs.^25^ Accordingly, the reduced QY in cNRs indicates that charge transfer between the PDA matrix and the NRs efficiently competes with the intrinsic radiative exciton recombination.

Next, the photocatalytic system was assembled by attaching to the cNRs several Rh catalysts as well as poly(ethylene glycol) (PEG) (MW = 5 kDa) chains as a stabilizer in a one-pot reaction via the formation of amide bonds between free amine groups of PDA and the carboxylic acid groups of the catalyst and the PEG chains. Grafting of PEG chains onto the cNRs was essential to achieve sufficient colloidal stability in buffer solutions and to prevent precipitation (see Figure S11). In the following, the PDA-coated nanorods functionalized with PEG and Rh catalysts are denoted as cNR-Rh-PEG.

The assembled cNR-Rh-PEG was characterized by energy-dispersive X-ray spectroscopy (EDX) (Figure 1G), which showed signals of the NR elements cadmium (3.1 keV), selenium (1.4 keV), and sulfur (2.3 keV). Signals for oxygen (0.5 keV) were assigned to the PDA coating, whereas carbon (0.3 keV) and nitrogen (0.4 keV) are present in both the PDA coating and the ligand of the Rh catalyst. The Rh signal at 2.7 keV indicates the successful functionalization of the cNRs with the Rh catalyst.

In addition, X-ray photoelectron spectroscopy (XPS) measurements were carried out to validate the EDX results and to assess whether the catalyst and PDA matrix were covalently conjugated. The XP survey and high-resolution Rh 3d spectra (Figure S12A,B) of cNR-Rh-PEG reveal the rhodium signal at 310.5 eV, indicating the successful covalent surface functionalization with the Rh catalyst. Furthermore, in the C 1s spectrum (Figure 1E,F), the shoulder at 286.6 eV was assigned to C–N/C–O bonds, which appears more intense for cNR-Rh-PEG compared with cNRs because of the addition of the PEG chains and the Rh catalyst. These findings are also supported by the high-resolution N 1s and O 1s spectra (Figure S12). Last, the absorption spectrum of cNR-Rh-PEG contains the characteristic absorption of the molecular Rh catalyst at 300 nm in addition to contributions of the NRs and PDA absorption (Figure S13).

Furthermore, the surface charges of cNRs, cNR-PEG, and cNR-Rh-PEG as determined by their ζ potentials were measured. Functionalization of the PDA shell with neutral PEG increases the ζ potential from −24.1 ± 0.5 mV (cNRs) to −14.2 ± 0.8 mV (cNR-PEG). The photocatalytic system cNR-Rh-PEG does not show any charged surface (ζ potential = 0.1 ± 0.2 mV), as the negatively charged cNR-PEG and the positively charged Rh complex neutralize each other. Therefore, it is unlikely for educts and products to stick to the surface of the photocatalytic system via electrostatic interactions during catalysis.

By utilizing a commonly applied thermal NAD^+^ reduction test,^30^ we further evaluated whether the successful immobilization procedure of [(ipphCOOH)Rh(Cp*)Cl]Cl afforded a catalytically active cNR-Rh-PEG system. Whereas for cNR-Rh-PEG formate-driven NADH formation was clearly observed, no NAD^+^ reduction was observed for the Rh-free system (see Figure S14). This clearly indicated that (i) the catalytic activity of the Rh complex is preserved following material integration and (ii) that the Rh center is spatially accessible by NAD^+^ as well as NaHCO_2_.

As a proof of principle for the light-driven activity of the system, the photocatalytic reduction of NAD^+^ was tested as well. cNR-Rh-PEG (10 μg/mL) and NAD^+^ (250 μM) were dissolved in demineralized water and irradiated with blue light (466 nm, 45–50 mW/cm^2^) at room temperature under an argon atmosphere. At this excitation wavelength, both the CdS rod and the PDA are directly photoexcited. The photocatalytic reduction of NAD^+^ to NADH was monitored by following the emission peak of NADH at 462 nm in the emission spectrum (λ_exc_ = 340 nm). The NADH concentration produced in the reaction medium was determined by calibration, and the efficiency of our photocatalytic system was analyzed by calculating the mass of NADH produced per unit mass of cNR-Rh-PEG (Figure 2B; for the calculation, see the SI).

**Figure 2:**
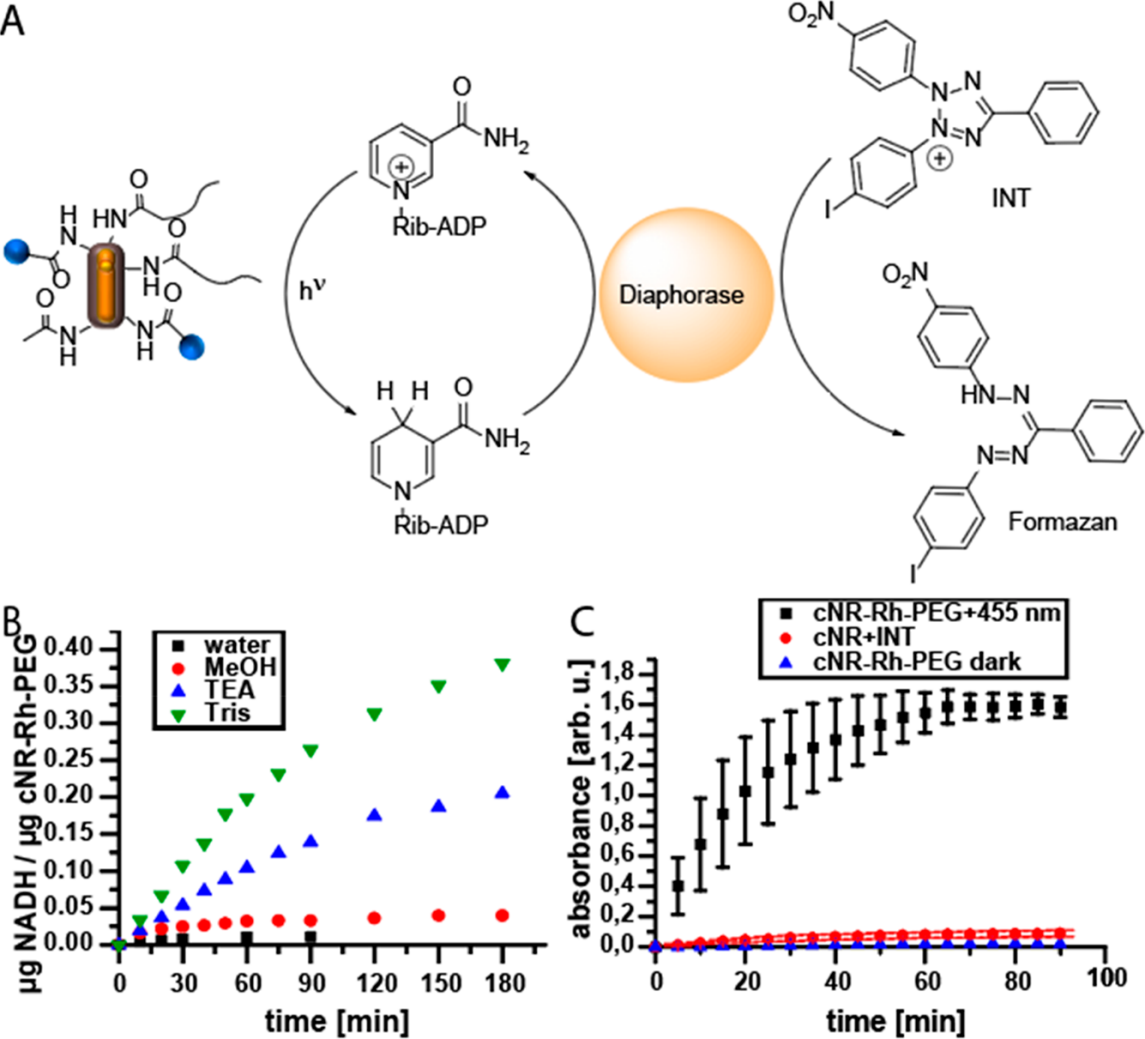
(A) Reaction scheme for the photocatalytic reduction of NAD^+^ with the coupled reduction of 2-(4-iodophenyl)-3-(4-nitrophenyl)-5-phenyl-2*H*-tetrazolium (INT) in the presence of diaphorase enzyme. (B) Produced mass of NADH per unit mass of the photocatalytic system (cNR-Rh-PEG) over time without or with MeOH, TEA, or Tris as a sacrificial agent, as determined from emission spectroscopy measurements. (C) Time evolution of the absorbance at 492 nm during the enzyme assay under irradiation and in the dark and for cNRs with INT under irradiation.

Irradiation of cNR-Rh-PEG in the presence of NAD^+^ afforded a minute increase in the emission detected at 462 nm, indicating the formation of NADH. To improve the photocatalyic efficiency, three different sacrificial agents were used: methanol (MeOH), triethylamine (TEA), and 2-amino-2-(hydroxymethyl)propan-1,3-diol (Tris). A 1:1 v/v MeOH/water mixture, water containing both TEA (0.12 M) and sodium dihydrogen phosphate (NaH_2_PO_4_) (0.1 M), and Tris-HCl buffer (25 mM, pH 7.5) were used as reaction media. In case of TEA as a sacrificial agent, the addition of NaH_2_PO_4_ was necessary to keep the pH acidic enough to guarantee stability of the cofactors (NAD^+^ and NADH). The addition of MeOH leads to a slight increase in NADH emission. In contrast, a mixture of nonfunctionalized cNRs and NAD^+^ did not produce any NADH under identical conditions (Figure S15). The addition of TEA and Tris further enhanced NADH production with cNR-Rh-PEG, in accordance with the fact that TEA is a well-known sacrificial reducing agent.^18^ Surprisingly, with Tris as the electron donor, 0.26 μg of NADH/μg of cNR-Rh-PEG was produced after 60 min of irradiation, which is about 26 times higher than without any sacrificial agent and still 2 times higher than when TEA was used as a sacrificial agent. The fact that Tris outperformed all of the other tested sacrificial agents could be related to an increased interaction of Tris with the PDA shell, on the basis of the strong Tris–PDA interactions found during PDA synthesis.^31^ Consequently, the amine and hydroxyl groups in Tris and the surface groups of PDA could in principle interact, resulting in coassembly and faster hole quenching, which is known to be the rate-limiting step in CdS-based photocatalysis.^8^ The corresponding signal of the time-dependent NADH production in the presence of Tris showed a linear increase for the first hour of irradiation and then gradually leveled off. To verify the stability of the system during photocatalysis EDX, measurement were performed before and after photocatalysis (Figure 1G,H). The EDX spectrum of the purified reaction solution shows that after 90 min of irradiation all of the elemental signals of the cNR-Rh-PEG can be observed, indicating that the system stays intact during catalysis. This is additionally supported by the absorbance measurements under the same conditions as for the photocatalytic NAD^+^ reduction in Tris buffer (Figure S17). The decrease in activation could be due to deactivation of the catalyst (though it is still bound to the system) or changes in the matrix composition.

Next, we evaluated whether the produced NADH could be used as a high-value chemical in further downstream chemical conversion. In biocatalysis, which is of emerging interest in industry because the use of enzymes facilitates low-energy, sustainable methods of producing high-value chemicals and pharmaceuticals potentially at lower costs, NADH is an important cofactor for these enzymatic reactions.^20^ Furthermore, enzymatic reoxidation of NAD^+^ can lead to higher activity of the light-sensitive system, as the reoxidation of NADH in its role as an electron donor can be avoided.^18^

Therefore, the optimized cNR-Rh-PEG was used for the regeneration of NADH to serve as a cofactor in a downstream chemical transformation with the enzyme diaphorase as biocatalyst (Figure 2A). Diaphorases transfer a hydride from NADH to another substrate molecule, such as 2-(4-iodophenyl)-3-(4-nitrophenyl)-5-phenyl-2*H*-tetrazolium (INT). The transformation of INT as the substrate to the respective formazan can be monitored by absorption spectroscopy at 492 nm, as confirmed in a model reaction with added NADH (Figure S18). In a one-pot reaction, cNR-Rh-PEG successfully reduced NAD^+^ to NADH, which subsequently transferred a hydride to INT in the presence of diaphorase. All of the reactions were performed in a quartz cuvette under a nitrogen atmosphere and irradiation with a blue LED (455 nm, 45 mW/cm^2^).

As shown in Figure 2C, a continuous increase in the absorption spectrum at 492 nm was observed when the reaction solution was irradiated with blue light (455 nm) but not in the dark. As control, cNRs without the catalyst were combined with INT in Tris-HCl and irradiated under the same reaction conditions used for the enzyme assay (for details, see the SI). Under these conditions, no absorbance increase at 492 nm was observed (Figure 2C), indicating the importance of the entire photocatalytic system for the successful downstream reaction. The apparent slow-down and eventual plateau in photocatalytic conversion after 60 min was caused by precipitation of the reaction product formazan when it reached its solubility limit in water. When dimethylformamide (DMF) was added to the reaction solution at different time points (after 5, 30, 60, and 90 min), the precipitated formazan was redissolved, and a linear increase in the absorbance over the entire reaction time was recorded (Figure S19), indicating continuous photocatalytic conversion of educts. However, DMF could not be added during photocatalysis to increase formazan solubility, as it leads to denaturation and loss in activity of the enzyme.

In summary, we have reported for the first time a system consisting of an inorganic semiconductor nanostructured photosensitizer with covalently attached molecular catalysts for NADH production. We designed a photocatalytic system consisting of CdSe@CdS nanorods coated with PDA and functionalized with Rh catalysts, which was prepared and characterized by applying state-of-the-art surface characterization techniques. By the application of a PDA shell, a molecular catalyst was successfully conjugated to the CdSe@CdS nanorods without the need for a thiol group, which is (i) usually required for such surface chemistry and (ii) may potentially act as a poison for, e.g., the Rh catalyst utilized herein.^32^ The assembly of a suspendable heterogeneous system was achieved by merging all of the photocatalytic entities within one nanosystem. Efficient photocatalytic activity was confirmed by the light-driven reduction of NAD^+^ to NADH, and this product was then used in a downstream chemical transformation, namely, the reduction of INT to the respective formazan by diaphorase with NADH as the cofactor. A direct comparison of the performance of this heterogeneous nanosystem with literature-known systems is not straightforward because in most of the comparable catalytic systems the photosensitizer and catalyst are homogeneously dissolved,^18,21^ leading to different reaction kinetics. Also, in the few examples that exist for heterocatalytic systems,^22^ the catalysts were not attached to the photosensitizer but remained molecularly dissolved, again resulting in important changes in the reaction kinetics relative to our system. It was not possible to calculate a turnover number for our system because of the detection limit for quantifying the amount of attached catalyst, which also makes it difficult to compare the efficiency of the hybrid cNR-Rh-PEG photocatalytic system to those of other systems from the literature.

The hybrid photocatalytic system reported herein combines photosensitizers, a redox-active polymer matrix, and molecular catalysts to produce functional chemicals such as NAD^+^. The PDA coating of CdSe@CdS nanorods serves as an ultrathin adherent organic layer that offers many promising features for integration into photocatalytic systems, such as easy functionalization with different catalysts that can catalyze various reactions. In this way, one could envision attaching two catalysts, one for each half-reaction, thereby combining reduction and oxidation reaction centers within one system, which could afford photocatalytic systems that function without the need for additional sacrificial agents.

## Supporting Information Available

Materials, additional experimental details, methods, instrumentation, additional TEM images, XPS spectra, absorption and emission spectra, and XRD profiles (PDF)

## Abbreviations

NAD^+^: β-nicotinamide adenine dinucleotide, oxidized form
NADH: β-nicotinamide adenine dinucleotide, reduced form
PDA: polydopamine
NRs: CdSe@CdS dot-in-rod nanostructures
cNRs: polydopamine-coated CdSe@CdS dot-in-rod nanostructures
cNR-Rh-PEG: polydopamine-coated CdSe@CdS dot-in-rod nanostructures functionalized with Rh catalyst and PEG
TEM: transmission electron microscopy
PEG: poly(ethylene glycol)
EDX: energy-dispersive X-ray spectroscopy
XPS: X-ray photoelectron spectroscopy
TEA: triethylamine
Tris: 2-amino-2-(hydroxymethyl)propan-1,3-diol
INT: 2-(4-iodophenyl)-3-(4-nitrophenyl)-5-phenyl-2*H*-tetrazolium
PL: photoluminescence
