# Supplementary material for: Rhodium-Complex-Functionalized and Polydopamine-Coated CdSe@CdS Nanorods for Photocatalytic NAD+ Reduction
Source: ACS Appl Nano Mater. 2021 Dec 2;4(12):12913–9. doi: 10.1021/acsanm.1c02994 (PMC8713362; doi:10.1021/acsanm.1c02994)
Supplement: Supplementary file 1 — an1c02994_si_001.pdf [file an1c02994_si_001.pdf]

Supporting Information for

Rhodium Complex-Functionalized and Polydopamine-Coated CdSe@CdS Nanorods for Photocatalytic NAD^+ Reduction

Marcel Boecker,[a] Mathias Micheel,[b] Alexander K. Mengele,[c] Christof Neumann,[d] Tilmann Herberger,[a] Tommaso Marchesi D'Alvise,[a] Bei Liu,[b,d] Andreas Undisz,[f,g] Sven Rau,[c] Andrey Turchanin,[d,e] Christopher V. Synatschke,[a] Maria Wächtler *[b,d,e] and Tanja Weil *[a]

[a] Department for Synthesis of Macromolecules, Max Planck Institute for Polymer Research, 55128 Mainz, Germany

[b] Leibniz Institute of Photonic Technology, 07745 Jena, Germany

[c] Institute of Inorganic Chemistry I, Ulm University, 89081 Ulm, Germany

[d] Institute of Physical Chemistry, Friedrich Schiller University Jena, 07743 Jena, Germany

[e] Abbe Center of Photonics (ACP), Albert-Einstein-Straße 6, 07745 Jena, Germany

[f] Institute of Materials Science and Engineering, Chemnitz University of Technology, 09125 Chemnitz, Germany

[g] Otto Schott Institute of Materials Research, Friedrich Schiller University Jena, 07743 Jena, Germany

Correspondence should be addressed to: Tanja Weil: weil@mpip-mainz.mpg.de

Maria Wächtler: maria.waechtler@leibniz-ipht.de

Table of Contents

1. General Methods 1

2. Synthesis 2

3. Instruments and techniques 7

4. Supporting Figures 10

5. SI References 17

1. General Methods

Material. All chemicals and solvents were purchased from Sigma Aldrich (Merck KGaA, Darmstadt, Germany) except octadecylphosphonic acid (ODPA) which was purchased from Carl Roth GmbH + Co. KG (Karlsruhe, Germany), acetic acid was purchased from VWR (Radnor, USA), 1-ethyl-3-(3-dimethylaminopropyl)carbodiimide (EDC) was purchased from G-Biosciences (St. Louis, USA) and 2-(4-iodophenyl)-3-(4-nitrophenyl)-5-phenyl-2*H*-tetrazolium (INT) and β-nicotinamide adenine dinucleotide hydride , oxidized (NAD^+) and reduced (NADH) form, were purchased from TCI EUROPE N.V. (Zwijndrecht, Belgium). Solvents of technical grade were utilized and were redistilled once using rotary evaporation prior to use for the synthesis of the catalyst. All reactions were performed under air if not otherwise stated. Synthesis of 1,10-phenanthroline-5,6-dione was performed as previously reported.[1]

Buffer solutions. Tris-HCl buffer (0.1 M, pH = 8.5 or 7.5) was prepared by dissolving Tris-(hydroxymethyl) aminomethane (121.14 g/mol, 0.005 mol) in 50 mL MilliQ water, and the pH was adjusted to 8.5 or 7.5 with 1 M HCl. Phosphate buffer (0.1 M, pH = 7) was prepared by dissolving sodium phosphate dibasic (anhydrous, 141.96 g/mol, 0.0482 mol) and sodium phosphate monobasic (anhydrous, 119.98 g/mol, 0.0018 mol) in 500 mL MilliQ water, and the pH was adjusted with 1 M HCl or 1 M NaOH if necessary. For all buffer solutions the pH was checked and adjusted using a pH-meter (Mettler Toledo MP220 pH meter).

2. Synthesis

Synthesis of CdSe seeds

The CdSe seed synthesis was adapted from a known procedure in literature.[2] 3.00 g trioctylphosphine oxide (TOPO), 0.28 g octadecylphosphonic acid (ODPA) and 0.06 g CdO were placed in a 25-mL-three-neck-flask. The reaction was performed under an inert atmosphere and constant stirring. The flask was heated to 80 °C to melt the chemicals and evacuated to remove water in the chemicals. Once no more gas emerged from the solution, the flask was heated to 120 °C and kept evacuated for 30 min. After that, the flask was purged with N_2. The flask was then heated to 320 °C, upon which the solution turned colorless due to the complexation of Cd-ODPA, and then cooled down to 120 °C. Then, a vacuum was applied to remove water, a side product of the complexation of Cd-ODPA; after gas formation in the reaction mixture stopped (at least 2 h), then, the flask was purged with N_2 and heated to 340 °C. Next, 0.058 g Se dissolved in 0.36 g trioctyl phosphine (TOP) were injected. After the injection, the heating was immediately stopped, and the flask was cooled down by N_2 airflow to accelerate the cooling speed. After the temperature of the mixture was cooled to 90 °C, 5 mL of toluene was injected into the mixture. The seeds were cleaned five times by centrifugation with 10 mL toluene in 10 mL methanol and then dissolved in toluene. The diameter of the CdSe seeds was determined from the energetic position of the absorption peak with the lowest energy[3] to be 5 nm.

Synthesis of the CdSe@CdS nanorods (NR)

The CdSe@CdS nanorods were synthesized according to a reported protocol in literature.[4] 3.35 g trioctylphosphine oxide (TOPO), 1.08 g octadecylphosphonic acid (ODPA), 0.207 g CdO, and 0.06 g *n*-propylphosphonic acid (PPA) were placed in a 25-mL-three-neck-flask. The reaction was performed under an inert atmosphere and constant stirring. The flask was heated up to 80 °C until the reaction mixture melted and evacuated to remove residual water from the mixture. Once gas formation stopped, the flask was heated to 120 °C and kept evacuated for 30 min. After that, the flask was flooded with N_2. The flask was then heated to 320 °C, upon which the solution turns colorless due to the complexation of Cd-ODPA, and then cooled down to 120 °C. Next, a vacuum was applied until gas formation in the reaction mixture stopped (at least 2 h) to remove water, which is a side product of Cd-ODPA complexation. Then, the flask was flooded with N_2 again and heated up to 340 °C. Next, 1.5 g TOP and 0.05 g sulfur dissolved in 0.60 g TOP were injected. After 20 s, 2 mg of CdSe seeds (diameter = 2.2 nm) dissolved in 0.5 g TOP were injected. The reaction was allowed to stir for 10 min until the color of the solution turned from red to orange. 5 mL toluene was injected into the mixture once the temperature dropped below the flashing point of toluene to stop solidification of the mixture. After precipitation with 10 mL methanol, rods were cleaned five times by centrifugation with 6 mL n-hexane, 2 mL nonanoic acid, and 2 mL octylamine in 10 mL methanol. Next, the size of the rods was excluded by centrifugation at 4200 rpm for 30 min with 10 mL toluene and 8 mL isopropanol to obtain rods with lengths of circa 50 nm. This was repeated two times in total. The NR were then dispersed in toluene for further investigation.

Ligand exchange with 11-mercaptoundecanoic acid (MUA)

The ligand exchange with MUA was performed according to a protocol in literature.[5] 250 mg MUA were dissolved in 25 mL methanol and tetramethylammonium hydroxide pentahydrate was added until the solution reached pH 11 (circa 200 mg). 20 mg NR (dried from its toluene solution under vacuum) were added into this mixture and stirred for 2 h. When the NR were fully dispersed, approximately 35 mL of toluene was added as non-solvent until NR precipitated. The mixture was then

centrifuged at 6000 rpm for 20 min, the supernatant discarded, and the precipitate was redispersed in degassed water.

Synthesis of 4-(1*H*-imidazo[4,5-*f*][1,10]phenanthrolin-2-yl)-benzoic acid (ipphCOOH)

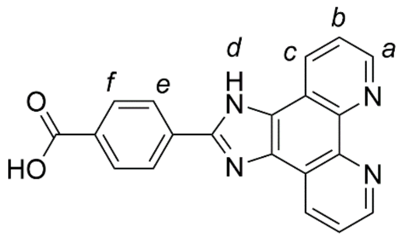

700 mg 1,10-phenanthroline-5,6-dione (3.34 mmol, 1 equiv.), 501 mg 4-formyl-benzoic acid (3.34 mmol, 1 equiv.) and 3.06 g ammonium acetate were placed in a 250 mL round bottom flask. After suspending the solids in 22 mL acetic acid, the mixture was refluxed for 30 min in a microwave setup using a power of 350 W. Upon cooling the mixture to room temperature, the formed yellow precipitate was filtered off and subsequently washed with H_2O and Et_2O. Afterwards the solid was dried under vacuum. Finally, 1.05 g (3.06 mmol, 93 %) of the desired yellow solid were obtained.

^{1}H-NMR (400 MHz, d_6-DMSO): δ = 13.80 (s, 1H_d), 9.08 – 8.99 (m, 2H_a), 8.89 (d, J = 8.1 Hz, 2H_c), 8.37 (d, J = 8.2 Hz, 2$H_{e/f}$), 8.16 (d, J = 8.2 Hz, 2$H_{e/f}$), 7.81 (dd, J = 7.7, 4.2 Hz, 2H_b).

^{13}C-NMR (101 MHz, d_6-DMSO): δ = 172.13, 167.07, 149.49, 148.08, 143.79, 133.69, 131.53, 130.10, 129.78, 126.20, 123.42, 21.13.

MS (APCI$^+$, [M] = $C_{20}H_{12}N_4O_2$): calcd. for $[M+H]^+$ 341.1, found: 340.9

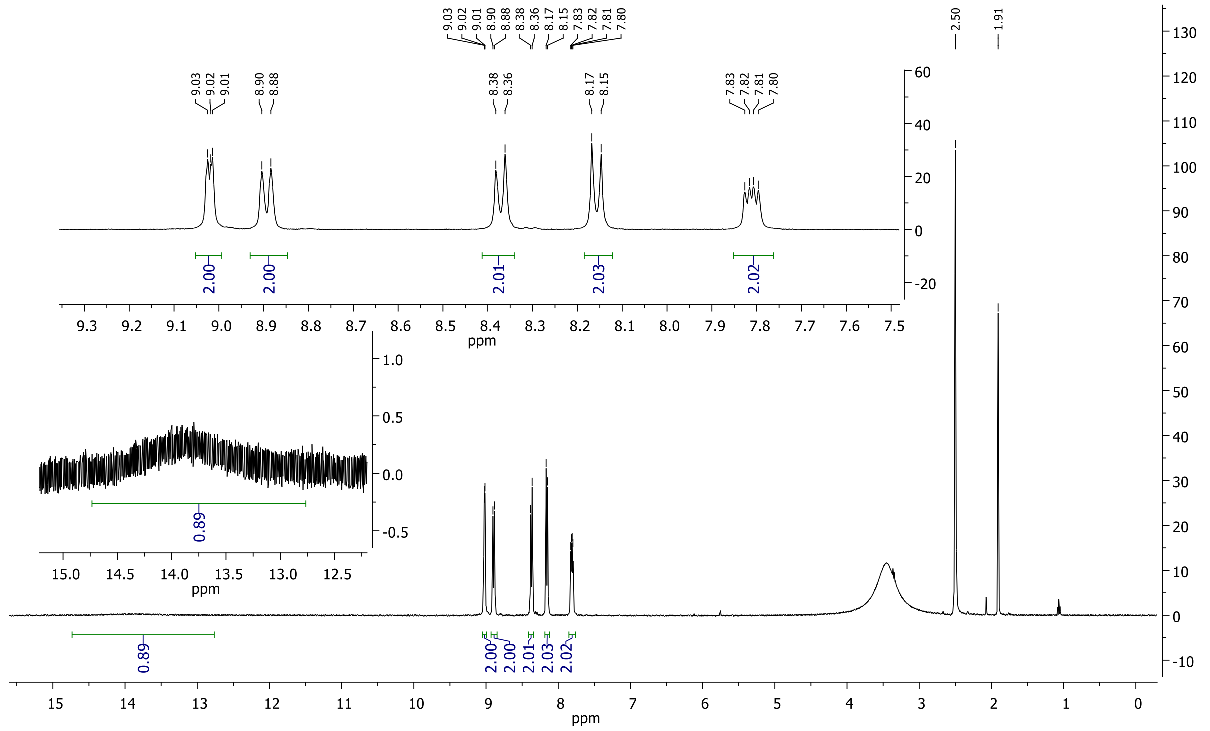

Figure S1: ^{1}H-NMR spectrum of ipphCOOH in d_6-DMSO. The large signal at 2.50 ppm and the broad peak at ca. 3.5 ppm are assigned to the H-containing solvent residual signal of d_6-DMSO and water, respectively.

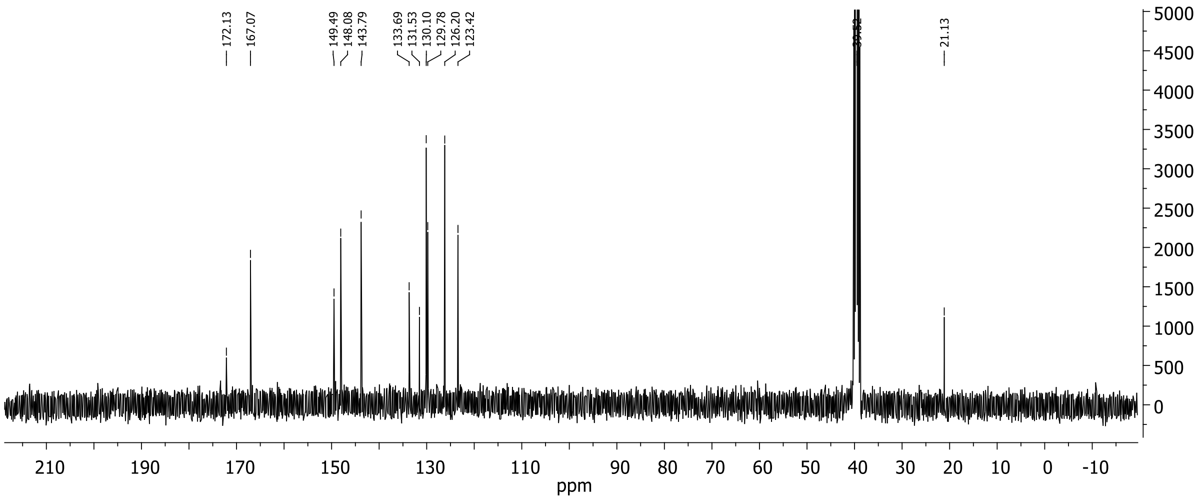

Figure S2: ^{13}C-NMR spectrum of ipphCOOH in d_6-DMSO. The large signal at ca. 40 ppm can be assigned to d_6-DMSO.

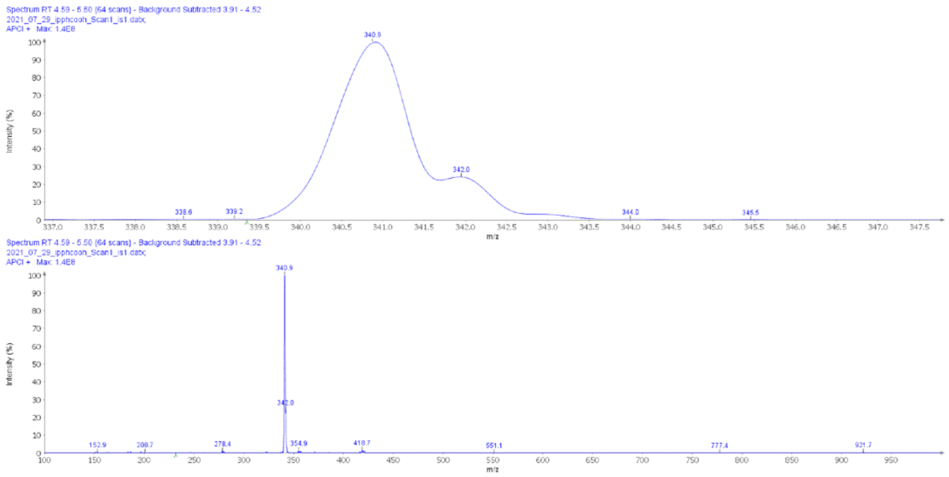

Figure S3: APCI mass spectrum (positive mode) of ipphCOOH. Bottom: full spectrum; top: expansion of the main peak.

<u>Synthesis of [(ipphCOOH)Rh(Cp*)Cl]Cl</u>

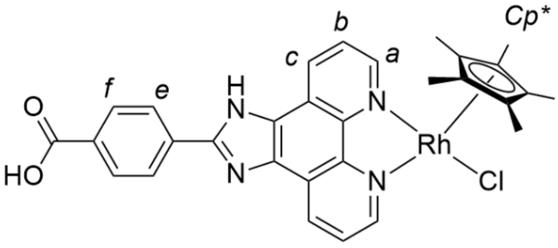

In a 50 mL round bottom flask, 19.0 mg (0.0307 mmol, 1 eq.) $[Rh(Cp^*)Cl_2]_2$ and 20.9 mg (0.0613 mmol, 2 equiv.) ipphCOOH were suspended in a 15 mL 1:1 (v:v) MeOH:DCM mixture. After ultrasonicating for 10 min, the mixture was stirred for 16 h at room temperature. After almost complete evaporation of the solvent, the desired compound was precipitated from the solution using Et_2O. After filtration, 37.8 mg (0.0582 mmol, 95%) of the compound were obtained as yellow solid.

^{1}H-NMR (400 MHz, d_4-MeOD): δ = 9.28 (dd, J = 5.2, 0.9 Hz, 2H_a), 9.09 (d, J = 8.1 Hz, 2H_c), 8.25 (d, J = 8.5 Hz, 2$H_{e/f}$), 8.17 (d, J = 8.5 Hz, 2$H_{e/f}$), 8.11 (dd, J = 8.1, 5.2 Hz, 2H_b), 1.82 (s, 15H_{Cp*}).

MS (ESI$^+$, [M] = $C_{30}H_{27}Cl_2N_4O_2Rh$): calcd. for $[M-Cl]^+$ 613.1, found 613.1; calcd. $[M-Cl-2H^++OH]^{2+}$ 314.0, found 314.0; $[M-Cl+H_3O^+]^{2+}$ 316.0, found 316.0.

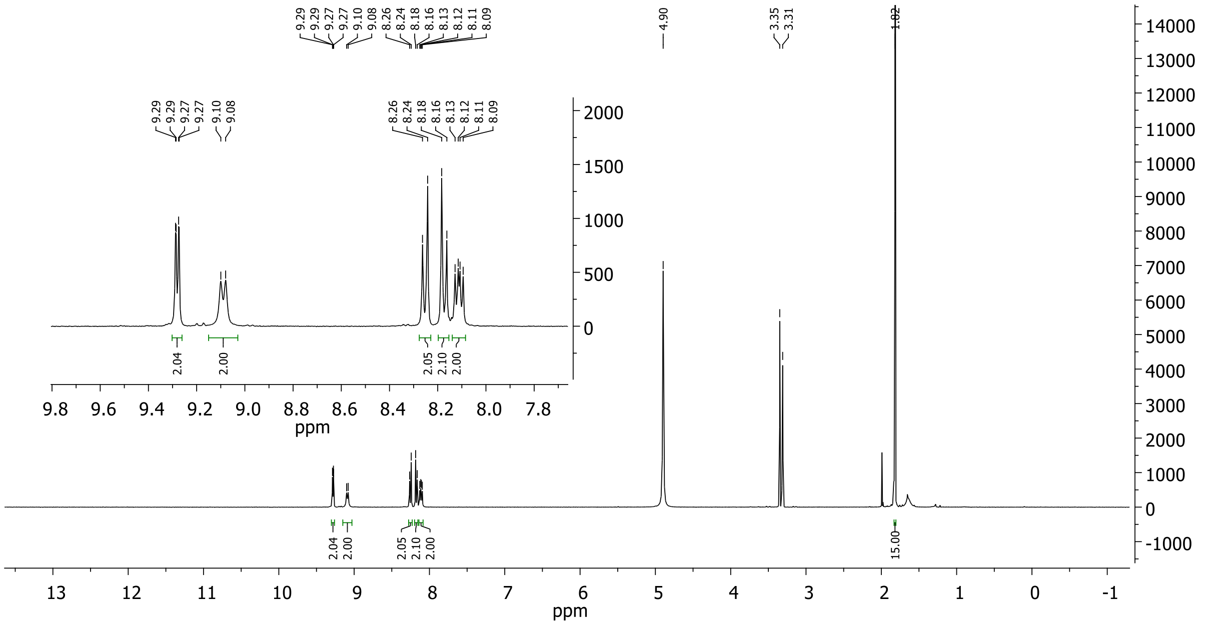

Figure S4: ^{1}H-NMR spectrum of [(ipphCOOH)Rh(Cp)Cl]Cl in d_4-MeOD. The large signals at 3.31 ppm, 3.34 ppm and 4.90 ppm are assigned to the H-containing solvent residual signal of d_4-MeOD, MeOH and water, respectively.*

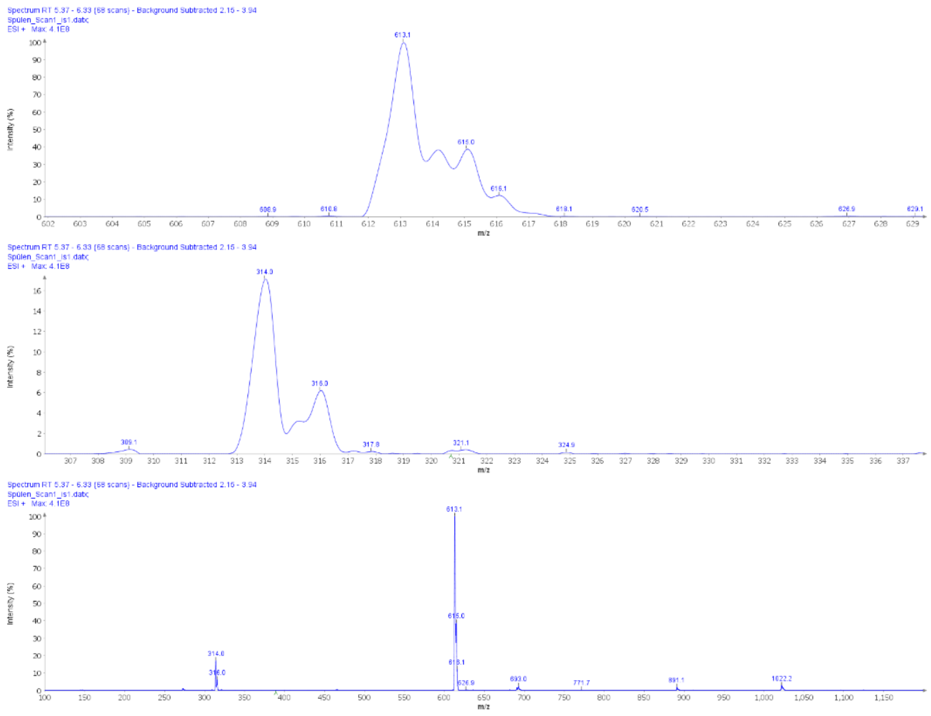

Figure S5: ESI mass spectrum (positive mode) of [(ipphCOOH)Rh(Cp)Cl]Cl. Bottom: full spectrum; center: expansion of the 314/316-peak; top: expansion of the main peak.*

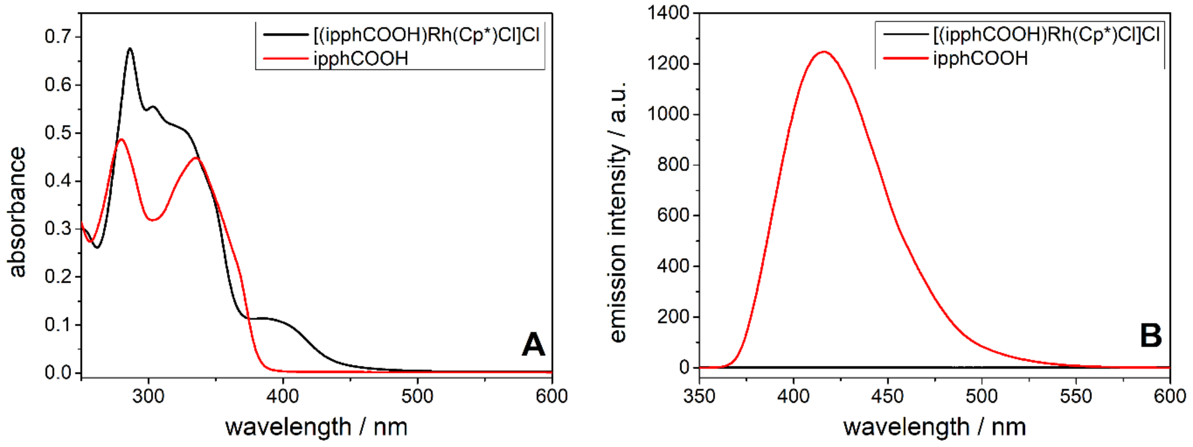

Figure S6: Panel A: UV-vis absorption spectra of 20 µM [(ipphCOOH)Rh(Cp)Cl]Cl (black line) and 20 µM ipphCOOH (red line) in MeCN under ambient conditions. Panel B: Emission spectra of 20 µM [(ipphCOOH)Rh(Cp*)Cl]Cl (black line) and 20 µM ipphCOOH (red line) in MeCN using the same solutions as for the spectra shown in panel A. λ_{exc} = 286 nm and λ_{exc} = 280 nm for [(ipphCOOH)Rh(Cp*)Cl]Cl (black line) and 20 µM ipphCOOH (red line), respectively.*

Polydopamine coating of the nanorods

50 µL of the nanorods solution (3 mg/mL) were diluted with 400 µL of Tris-HCl buffer (0.1 M, pH = 8.5) in an 1.5 mL Eppendorf tube and 50 µL of dopamine solution (1 mg/mL in MilliQ water) were added. The Eppendorf tube was wrapped in aluminum foil to prevent light exposure. Then, the reaction solution was vortexed for 24 h at 1000 rpm at room temperature. For purification, the reaction solution was filtered through a centrifuge filter at 4000 g to remove small molecule residuals like free dopamine. The coated nanorods remained in the filter and were redispersed in MilliQ water. The filtration was repeated twice. As centrifuge filters Millipore centrifugal filters units (Amicon Ultra – 0.5 mL, Ultracel – 100k) with a cut-off of 100 kDa and a centrifuge (Heraeus Fresco 2, Thermo Fisher Scientific) was used. For further functionalization the remaining PDA-coated nanorods were redispersed in phosphate buffer (0.1 M, pH = 8.5) while they were redispersed in MilliQ for storing.

Functionalization of coated nanorods with catalyst and PEG

For functionalization 1-ethyl-3-(3-dimethylaminopropyl)carbodiimide (EDC, 1.2 eq.), *N*-hydroxysuccinimide (NHS, 2 eq.), methoxypolyethylene glycol 5,000 acetic acid (PEG, 0.03 eq.) and 0.1 mL of [(ipphCOOH)Rh(Cp*)Cl]Cl dissolved in methanol (3 mg/mL) were added to 0.5 mL of PDA-coated nanorods in phosphate buffer (0.1 M, pH = 7) in an Eppendorf tube. The Eppendorf tube was wrapped in aluminum foil to prevent light exposure and the solution was vortexed for 24 h at 1000 rpm at room temperature. For purification, the reaction solution was filtered three times through a centrifuge filter at 4000 g to remove small molecules while the functionalized nanorods remained in the filter and were redispersed in MilliQ water. The same centrifuge filters as for the coated nanorods were used.

3. Instruments and techniques

NMR-Spectroscopy: ^{1}H-NMR (400 MHz) spectra were recorded on a Bruker Avance 400 MHz at room temperature.

^{13}C-NMR (126 MHz) spectra were recorded on a Bruker Avance 500 MHz at room temperature. The shift values are given in ppm and are referenced to the corresponding solvent residual peaks (4.79 ppm (singlet, ^{1}H-spectra) for D_2O, 3.31 ppm (quintet, ^{1}H-spectra) or 49.00 ppm (septet, ^{13}C-spectra) for d_4-MeOD and 2.50 ppm (quintet, ^{1}H-spectra) or 39.52 ppm (septet, ^{13}C-spectra) for d_6-DMSO. Peak multiplets are given as singlets (s), doublets (d), doublets of doublets (dd), or multiplets (m), respectively.

Steady-state absorption spectroscopy: Absorption spectra of nanorods (both bare and coated) were recorded in a quartz cell (d = 1 cm) using a JASCO V780 UV-Visible/NIR spectrophotometer (JASCO GmbH, Pfungstadt, Germany). All measurements were performed in a wavelength range of 300 nm to 700 nm. A cuvette with pure solvent was always measured as a reference.

Steady-state photoluminescence spectroscopy: Photoluminescence spectra of nanorods (both bare and coated) were recorded in a quartz cell (d = 1 cm) using an FLS980 photoluminescence spectrometer (Edinburgh Instruments Ltd., Livingston, UK) in a 90° geometry. An excitation wavelength of 450 nm was used to record photoluminescence spectra covering a wavelength range of 500 nm to 650 nm. The optical density of the dispersions was set to 0.05 to avoid inner filter effects and reabsorption of photoluminescence. Absolute photoluminescence quantum yields of bare nanorods were recorded at an excitation wavelength of 450 nm using a barium sulfate coated integrating sphere.[6, 7] The integrating sphere was mounted on the fluorimeter with the entry and output ports of the sphere located in 90° geometry from each other in the plane of the spectrometer. Photoluminescence was recorded from 425 to 700 nm. The colloidal samples were held in a five-face transparent quartz cuvette located in the center of the integrating sphere and the optical density at the excitation wavelength was adjusted to 0.1. As a reference sample, a cuvette filled with pure water was recorded under identical conditions. The quantum yield of coated nanorods were estimated by measuring both bare and coated nanorods under similar conditions, i.e., same excitation parameters and a similar absorbance at the excitation wavelength, without using the integrating sphere.

Thermal NAD^+ reduction: In order to test for the catalytic activity of immobilized Rh-catalyst, a 10 µg/mL concentrated suspension of either cNR-Rh-PEG or cNR-PEG in 50 mM aqueous $NaHCO_2$ was prepared in an argon filled glovebox and transferred into a sealable cuvette filled with 2.5 mL of the reaction solution. The solution, which additionally contained 250 µM NAD^+, was heated to 45 °C using a water bath. After certain time intervals the reaction progress was analyzed using emission spectroscopy on a JASCO FP-8500 fluorescence spectrometer (λ_{exc} = 340 nm).

Irradiation setup for photocatalysis: For the NAD^+ reduction, 250 µM NAD^+ and 10 µg/mL of the PDA-coated nanorods functionalized with the Rh catalyst and PEG were dissolved in the respective solvent. As solvents either demineralized water, a water methanol mixture (1:1), water containing 0.12 M trimethylamine (TEA) and 0.1 M NaH_2PO_4 or Tris-HCl buffer (25 mM, pH = 7.5) were used. All reactions were performed with 2.5 mL of the reaction solution at room temperature inside gas-tight quartz glass cuvettes (d = 10.0 mm, Hellma) under argon atmosphere. The reactions were irradiated for 180 minutes. The irradiation setup consisted of a custom-made reactor and one blue light emitting LED-stick (λ_{max} = 466 nm, 45-50 mW/cm^2) in the center of the reactor on which the cuvettes were placed.[8] The photocatalysis runs were analyzed via UV-vis spectroscopy on a JASCO V-670 UV-vis-NIR spectrophotometer or emission spectroscopy on a JASCO FP-8500 fluorescence spectrometer.

Quantification of produced NADH: For determination of the amount of NADH produced during photocatalysis, a calibration curve using commercially available NADH (Sigma Aldrich, 97% purity) was

recorded as follows: Samples of different NADH concentrations (0 µM, 2.5 µM, 5.0 µM, 10.0 µM, 15.0 µM, 20.0 µM, 25.0 µM and 30.0 µM) were prepared in 2.5 mL deionized water under ambient conditions. Emission spectra were recorded using λ_{exc} = 340 nm. The intensity of the emission maximum at 462 nm was plotted against the molar concentration of NADH to obtain the required calibration curve. (see Figure S18). The known masses of NADH were divided by the mass of the photocatalytic system (10 µg/mL) and plotted against the measured intensities (see Figure S21). The data was fitted with a square function giving

$$y = 2 \cdot 10^{-8} x^2 + 0.0004x$$

with x being the intensity and y the mass NADH per mass of photocatalytic system. The produced mass of NADH per mass of the photocatalytic system was calculated using the given equation and the measured intensities.

Irradiation setup for enzyme assay: The assay was performed at room temperature inside a quartz glass cuvette (100 µL, 10 x 2 mm, Hellma Analytics) under nitrogen atmosphere. The 200 µL reaction solution contained 2 U of the diaphorase, the PDA-coated nanorods functionalized with the Rh catalyst and PEG (10 µg/mL), 0.5 mM 3-(4-Iodophenyl)-2-(4-nitrophenyl)-5-phenyl-2*H*-tetrazol-3-ium chloride (INT), 2.5 mM NAD^+ in 200 µL Tris-HCl buffer (25 mM, pH = 7.5). The photocatalysis runs were analyzed via UV/Vis absorbance measurements with an Avantes Starline AvaSpec-2048 with an AvaLight-DH-S-BAL as light source. The irradiation setup consisted of a custom-made cuvette holder with a shutter (Thorlabs SHB1T, series shutter controller) with a blue LED (λ_{max} = 455 nm, 45 mW/cm^2, Thorlabs) and the optical fibers (ThorLabs M114L01 600µm 0.22 NA) attached to the spectrophotometer and the cuvette holder (in a 90° angle to the blue LED). The shutter was controlled by a potentiostat (Metrohm Autolab, type: PGSTAT204) to block the light of the LED while measuring the UV/Vis. For the experiments with added DMF, 20 µL of DMF were added after the different time intervals to the reaction solution.

Energy-dispersive X-ray spectroscopy (EDX): The EDX spectrum was recorded on an Hitachi SU8000 scanning electron microscope (Hitachi High-Technologies Europe GmbH, Krefeld) with a Bruker Quantax EDX device. For the measurements a primary energy of 8 keV, a tilt angle of 0°, a take off angle of 30° and as detector a Bruker XFlash 5010 (fifth generation, 10 mm^2 detector area) was used. The data was analyzed using the Bruker Quantax Esprit 2.3 software.

Transmission electron microscopy (TEM): TEM images of nanorods after synthesis and before ligand exchange were recorded in scanning mode (STEM) using a JEM-ARM200F NEOARM (Jeol) operating at 80 kV, equipped with spherical aberration corrector, bright field (BF), annular bright field (ABF) and annular dark field (ADF) detectors. Several images were recorded and 640 particles were analyzed regarding their length and width using FIJI (ImageJ v. 1.53c).[9] The TEM images for determination of the coating thickness and after the treatment with $HAuCl_4$ were recorded with a Tecnai F20 (Field Electron and Ion Company, FEI) using an accelerating voltage of 200 kV.

X-ray photoelectron spectroscopy (XPS): XPS was performed in an ultra-high-vacuum ($<2 \times 10^{-10}$ mbar) multiprobe system (Scienta Omicron) using a monochromatized X-ray source (Al K_α, 1486.7 eV) and an electron analyzer (Argus CU) with a spectral resolution of 0.6 eV. The spectra were calibrated using the Au $4f_{7/2}$ peak (84.0 eV) and the Si 2p peak (SiO_2, 103.6 eV), respectively and fitted using Voigt functions (30:70) after background subtraction.

Electrospray ionization mass spectrometry (ESI-MS): measurements were performed on an Advion expression-LCMS with a single quadrupole mass analyzer and an electron multiplier with a high energy conversion dynode detector. The unit mass resolution of 0.5 to 0.7 *m/z* (FWHM) is provided over the range of 0 – 2000 *m/z* units and the accuracy is 0.1 *m/z*.

Atmospheric Pressure Chemical Ionization (APCI): measurements were performed on an Advion expression-LCMS with a single quadrupole mass analyzer and an electron multiplier with a high energy conversion dynode detector. The unit mass resolution of 0.5 to 0.7 *m/z* (FWHM) is provided over the range of 0 – 2000 *m/z* units and the accuracy is 0.1 *m/z*.

X-ray diffraction analysis (XRD): measurements were performed on a Rigaku SmartLab using a Cu K-α anode with $\lambda = 1.540$ Å. The material was drop-casted on a zero background Si substrate and dried at 50 °C.

Zeta-potential: 60 µL of cNR, cNR-PEG or cNR-Rh-PEG (0.2 mg/mL) were diluted to 1 mL in an aqueous solution of 1 mM KCl. The dilution ratio was adjusted, if the count rate was not sufficient. The zeta potential was derived from the electrophoretic mobility of the particles and measured using a Zeta Nanosizer ZS (Malvern Instrument) with 1 mL disposable folded capillary cells (Zeatasizer Nano series, Malvern). Each measurement was performed in triplicates.

4. Supporting Figures

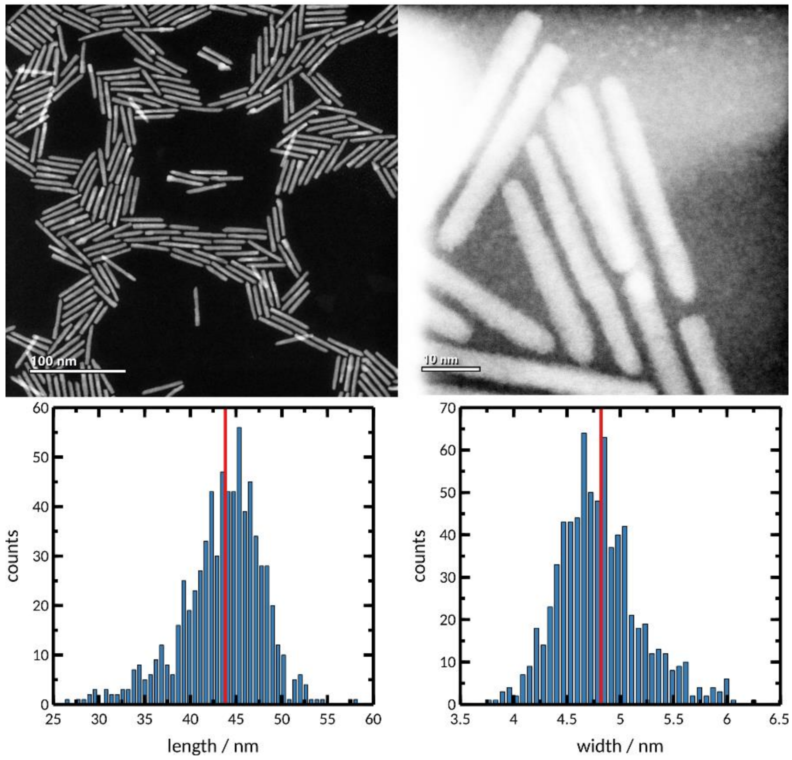

Figure S7: Representative STEM-ADF images of the nanorods used and histograms of their length and width. 640 individual particles were analyzed, yielding an average length of 43.8 ± 5.8 nm and width of 4.8 ± 0.4 nm for the NR.

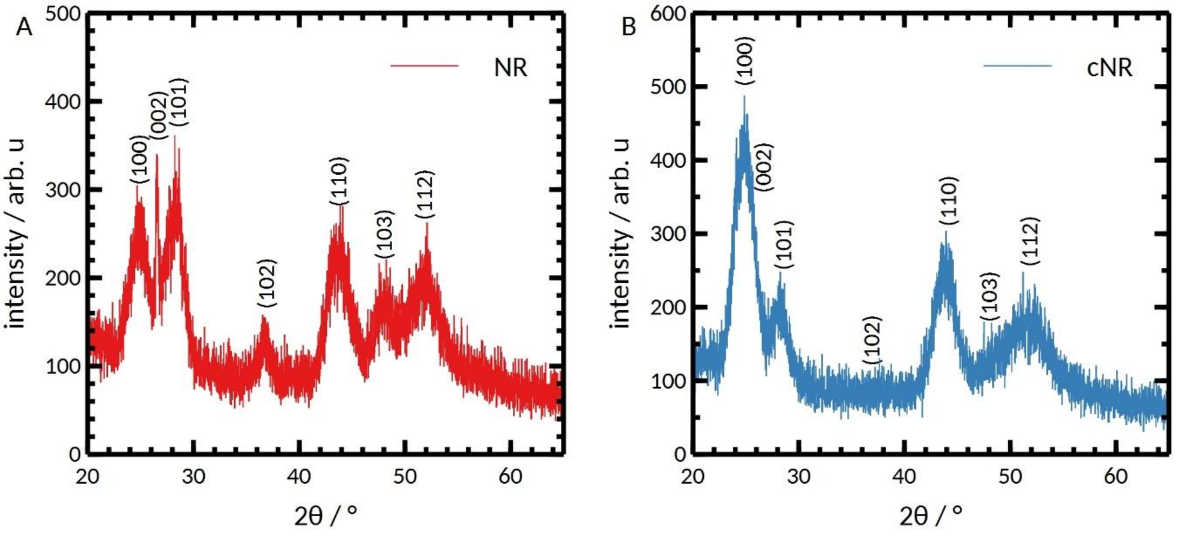

Figure S8: XRD Spectra of the non-functionalized NR and cNR. For NR, the XRD peaks have been assigned to specific crystal facets.

The XRD pattern (Figure S8) of NR shows (102) and (103) reflections at 36.6° and 47.9°, respectively, characteristic for the wurtzite structure. Additionally, the intense (002) reflection is characteristic for CdSe@CdS NR obtained from the seeded-growth method due to the preferred growth along this facet.[4] The XRD pattern of cNR is qualitatively the same, but some reflections, in particular the (102) and (103) reflections, are less intense, while the (100) reflection appears more pronounced.

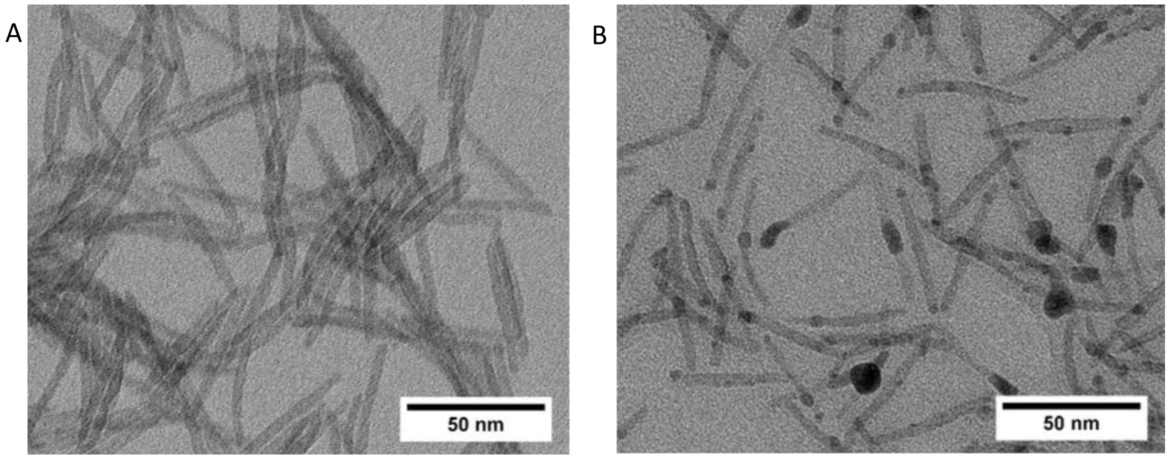

Figure S9: TEM images of bare (A) and coated (B) nanorods after treatment with HAuCl4. Au nanoparticles in the cNR image appear due to reduction by the PDA shell.

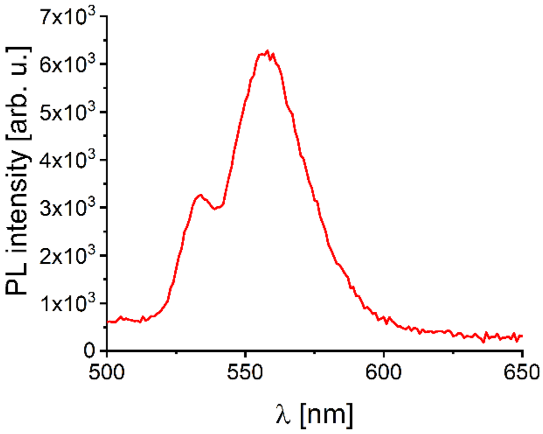

Figure S10: Photoluminescence spectrum of cNR (λ_{ex}= 450 nm) of coated nanorods in water. Photoluminescence of cNR has an estimated photoluminescence yield in the order of 0.001. The shoulder at 530 nm stems from the raman scattering of water.

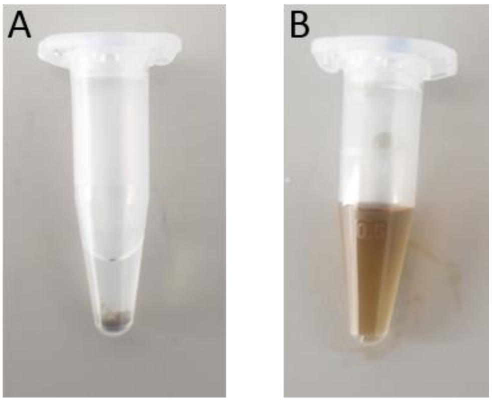

Figure S11: Photograph of (A) cNR functionalized only with Rh catalyst and (B) the cNR functionalized with Rh catalyst and PEG.

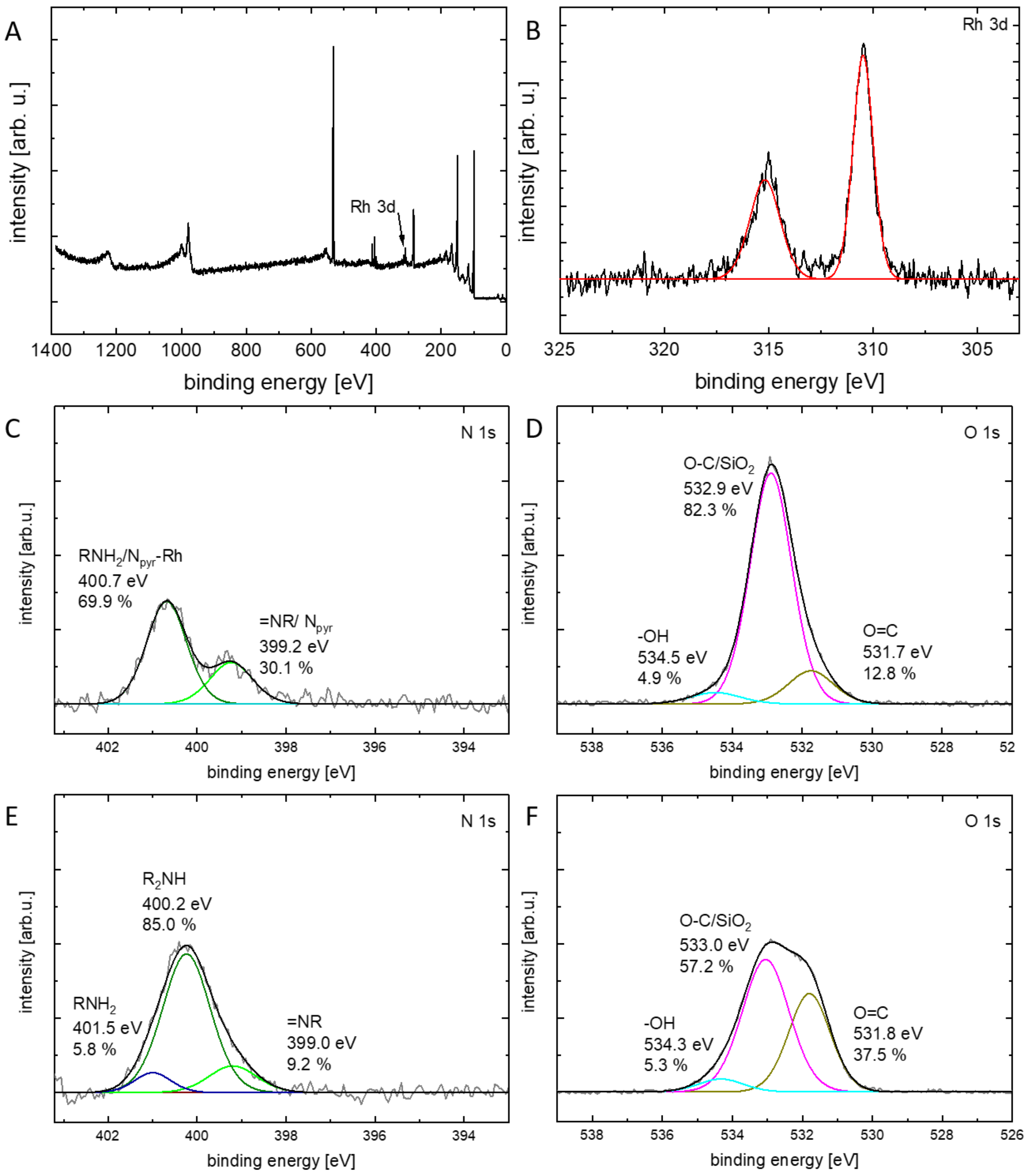

Figure S12: XP survey spectrum of the photocatalytic system (A), the high-resolution Rh 3d (B), N 1s (C) and O 1s (D) XP spectra of the photocatalytic system and the high-resolution N 1s (E) and O 1s (F) XP spectra of the cNR.

The observed features in the high-resolution N 1s (E) and O 1s (F) spectra of the cNR match well to the literature of polydopamine films.[10] After the Rh-catalyst and the PEG are attached to the cNR, the spectra change slightly. The nitrogen atoms from imidazole, amide and pyridine in the Rh catalyst contribute to the species at lower binding energy, whereas both nitrogen atoms from the pyridine groups coordinated with the Rh atoms can be ascribed to the signal at higher binding energies (see Figure S13 C). In the O 1s spectrum (D), the component ascribed to O-C and SiO_2 increased due to two different reasons. On the one hand, the addition of PEG leads to a higher amount of O-C bonds and on the other hand the oxygen signal from the SiO_2 substrate is stronger in comparison to (F), as confirmed by the more intense Si 2p signal (not shown).

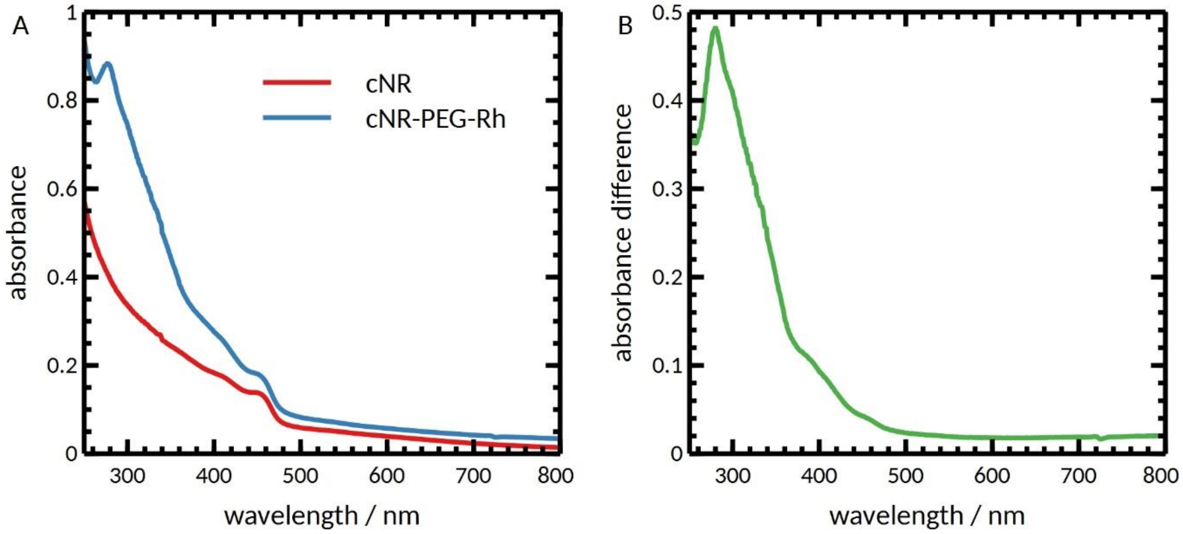

Figure S13: (A) Comparison of the absorption spectra of cNR and cNR-Rh-PEG. (B) The difference spectrum of cNR and cNR-PEG-Rh closely matches the absorption spectrum of the Rh catalyst measured in acetonitrile (Figure S6).

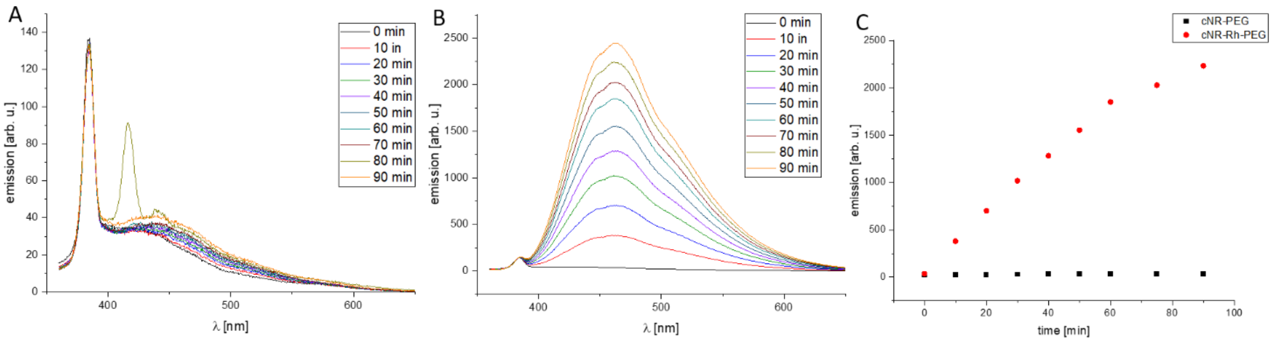

Figure S 14: Emission spectra of the thermal catalysis with (A) cNR-PEG or (B) cNR-Rh-PEG and (C) the emission at 463 nm over time for the thermal catalysis with and without the attached Rh catalyst to the system.

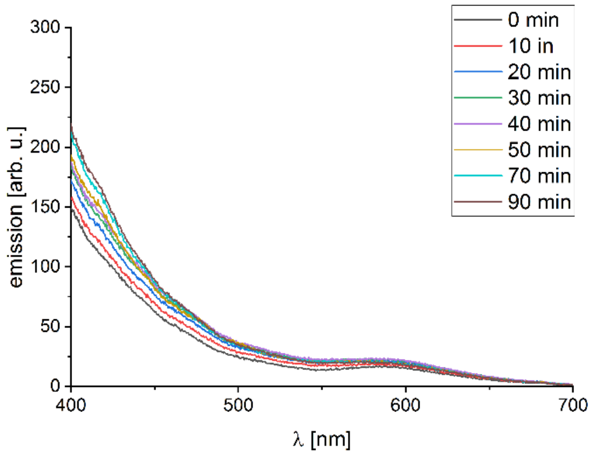

Figure S15: Emission spectra (λ_{exc} = 340 nm) of the reaction solution containing cNR with NAD^+ in a water methanol mixture (V:V = 1:1) under argon atmosphere and irradiation with blue light (466 nm, 45-50 mW/cm^2).

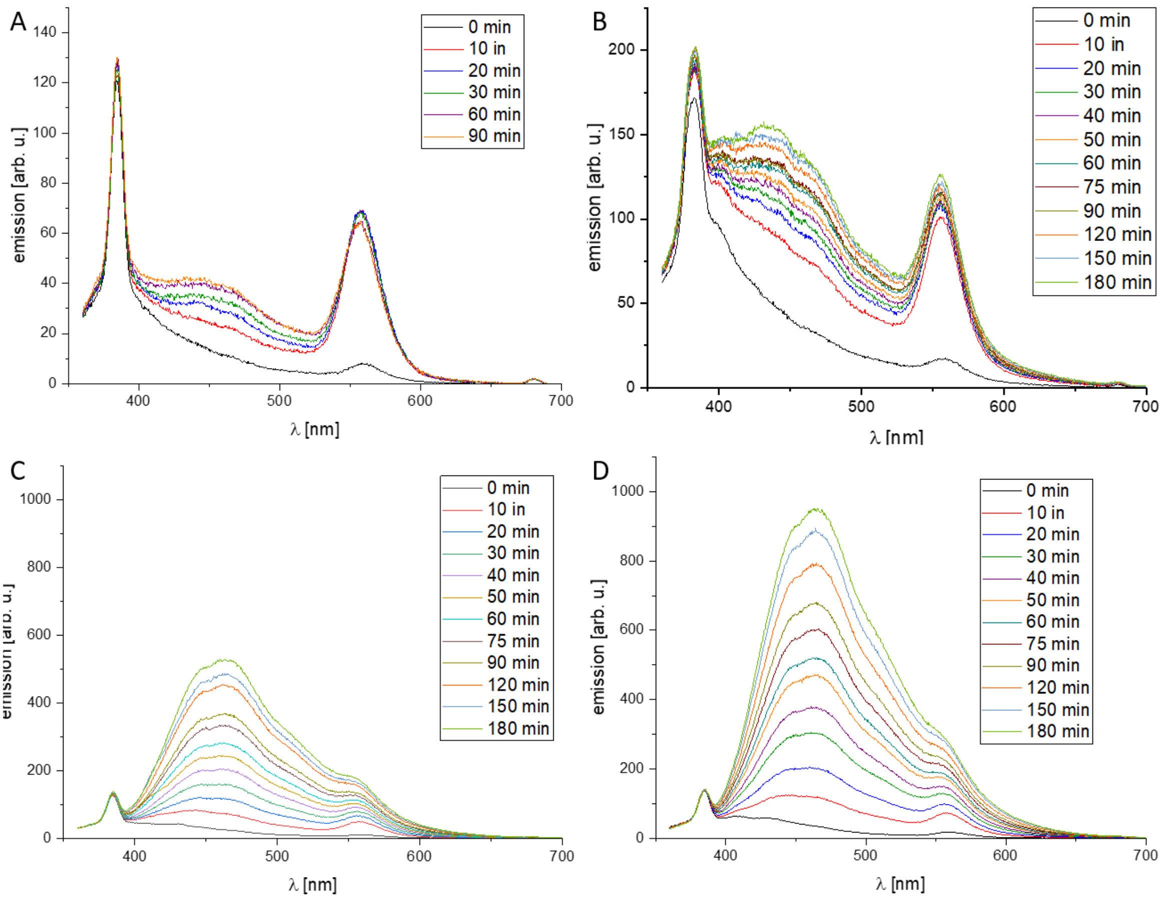

Figure S16: Emission spectra of the photocatalytic NAD^+ reduction with cNR-Rh-PEG in (A) water, (B) water:MeOH (V:V = 1:1), (C) water with TEA and NaH_2PO_4 and (D) Tris- buffer (25 mM, pH = 7.5). In any case, the system was kept under argon and irradiation was performed using blue light (466 nm, 45-50 mW/cm²).

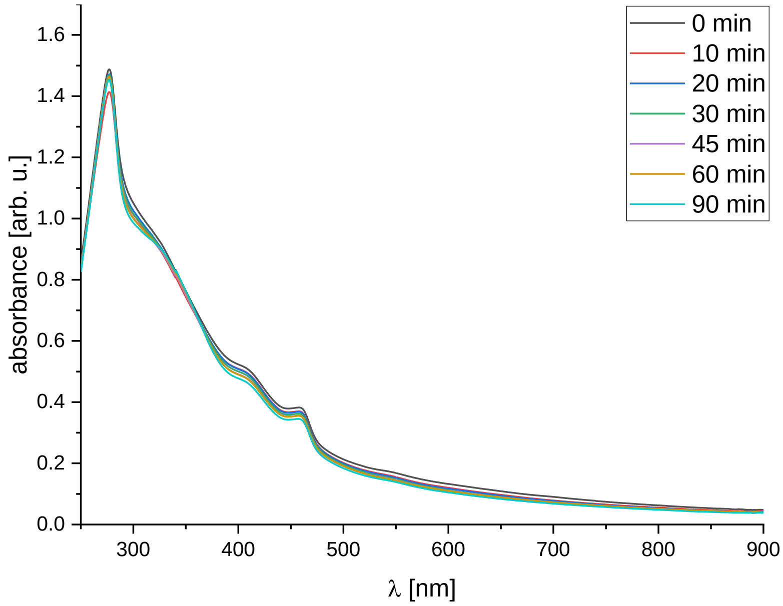

Figure S17: Absorbance spectra of the cNR-Rh-PEG during photocatalytic NADH production in Tris buffer.

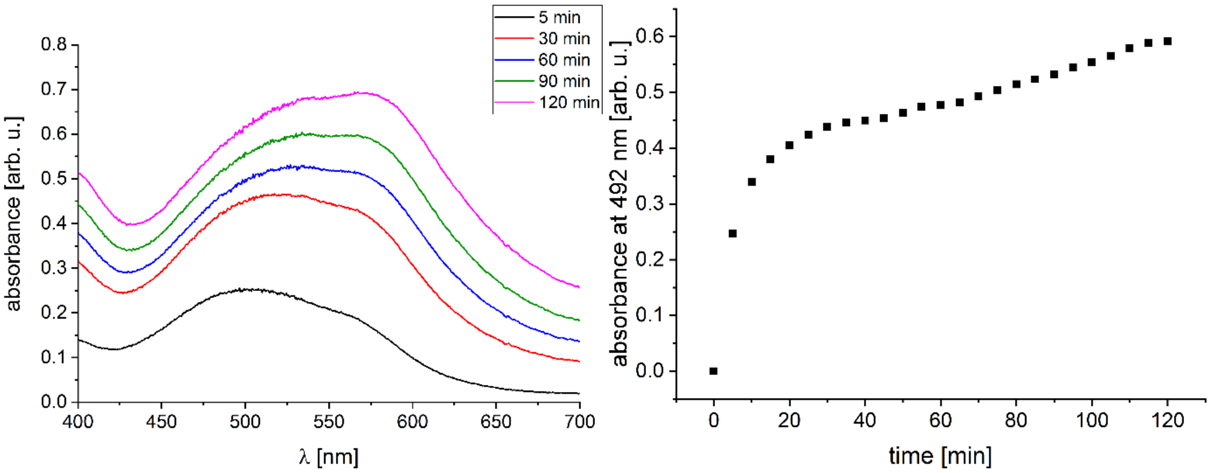

Figure S18: Absorption spectrum of the enzyme assay with added NADH (left), absorption at 492 nm over time (right).

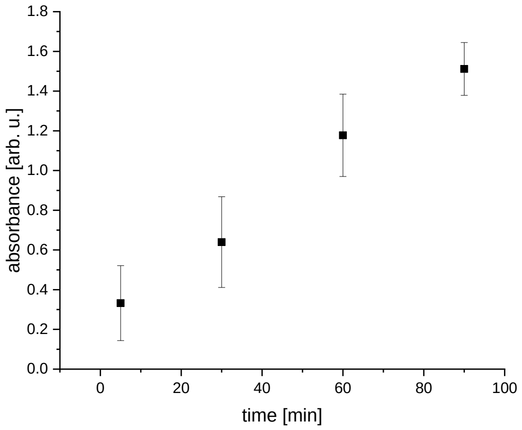

Figure S 19: Absorption at 492 nm of the enzyme assay with added DMF after 5 min, 30 min, 60 min or 90 min.

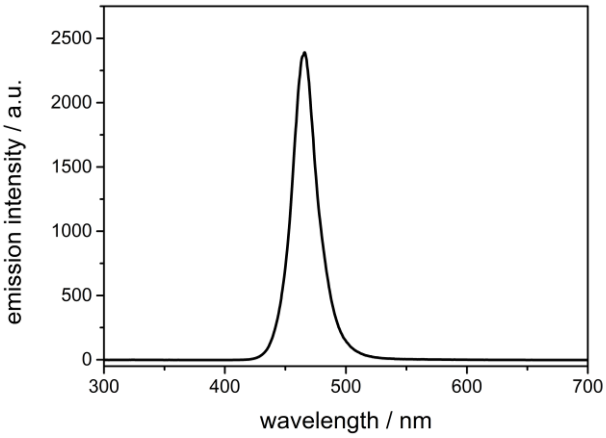

Figure S20: Emission profile of the utilized LED stick (λ_{max} = 466 nm, FWHM = 23 nm).

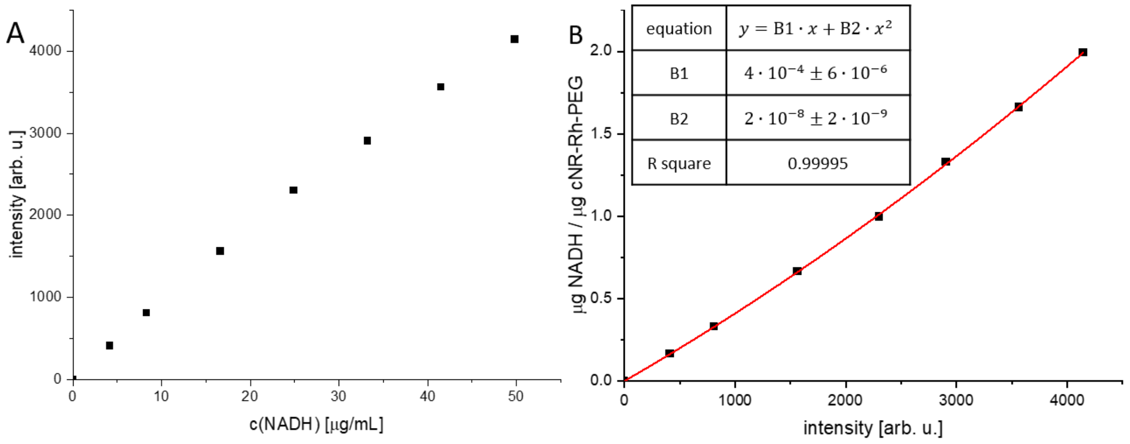

Figure S21: (A) Maxima of different emission spectra with different NADH concentrations for calibration. (B) Fitted intensities from the measurements in 2.5 mL plotted against µg NADH per µg photocatalytic system (PCS).

5. SI References

1. Yamada, M.; Tanaka, Y.; Yoshimoto, Y.; Kuroda, S.; Shimao, I., Synthesis and Properties of Diamino-Substituted Dipyrido [3,2-a: 2′,3′-c]phenazine. *Bulletin of the Chemical Society of Japan* **1992,** *65* (4), 1006-1011.
2. Carbone, L.; Nobile, C.; De Giorgi, M.; Sala, F. D.; Morello, G.; Pompa, P.; Hytch, M.; Snoeck, E.; Fiore, A.; Franchini, I. R.; Nadasan, M.; Silvestre, A. F.; Chiodo, L.; Kudera, S.; Cingolani, R.; Krahne, R.; Manna, L., Synthesis and micrometer-scale assembly of colloidal CdSe/CdS nanorods prepared by a seeded growth approach. *Nano Lett* **2007,** *7* (10), 2942-50.
3. Jasieniak, J.; Smith, L.; van Embden, J.; Mulvaney, P.; Califano, M., Re-examination of the Size-Dependent Absorption Properties of CdSe Quantum Dots. *The Journal of Physical Chemistry C* **2009,** *113* (45), 19468-19474.
4. Talapin, D. V.; Nelson, J. H.; Shevchenko, E. V.; Aloni, S.; Sadtler, B.; Alivisatos, A. P., Seeded growth of highly luminescent CdSe/CdS nanoheterostructures with rod and tetrapod morphologies. *Nano Lett* **2007,** *7* (10), 2951-2959.
5. Amirav, L.; Alivisatos, A. P., Photocatalytic Hydrogen Production with Tunable Nanorod Heterostructures. *J Phys Chem Lett* **2010,** *1* (7), 1051-1054.
6. Geissler, D.; Wurth, C.; Wolter, C.; Weller, H.; Resch-Genger, U., Excitation wavelength dependence of the photoluminescence quantum yield and decay behavior of CdSe/CdS quantum dot/quantum rods with different aspect ratios. *Phys Chem Chem Phys* **2017,** *19* (19), 12509-12516.
7. Porres, L.; Holland, A.; Palsson, L. O.; Monkman, A. P.; Kemp, C.; Beeby, A., Absolute measurements of photoluminescence quantum yields of solutions using an integrating sphere. *J Fluoresc* **2006,** *16* (2), 267-72.
8. Pfeffer, M. G.; Schäfer, B.; Smolentsev, G.; Uhlig, J.; Nazarenko, E.; Guthmuller, J.; Kuhnt, C.; Wächtler, M.; Dietzek, B.; Sundström, V.; Rau, S., Palladium versus platinum: the metal in the catalytic center of a molecular photocatalyst determines the mechanism of the hydrogen production with visible light. *Angew Chem Int Ed Engl* **2015,** *54* (17), 5044-8.
9. Schindelin, J.; Arganda-Carreras, I.; Frise, E.; Kaynig, V.; Longair, M.; Pietzsch, T.; Preibisch, S.; Rueden, C.; Saalfeld, S.; Schmid, B.; Tinevez, J. Y.; White, D. J.; Hartenstein, V.; Eliceiri, K.; Tomancak, P.; Cardona, A., Fiji: an open-source platform for biological-image analysis. *Nat Methods* **2012,** *9* (7), 676-82.
10. Rella, S.; Mazzotta, E.; Caroli, A.; De Luca, M.; Bucci, C.; Malitesta, C., Investigation of polydopamine coatings by X-ray Photoelectron Spectroscopy as an effective tool for improving biomolecule conjugation. *Applied Surface Science* **2018,** *447*, 31-39.
